# Relationships between climate and year-to-year variability in meningitis outbreaks: A case study in Burkina Faso and Niger

**DOI:** 10.1186/1476-072X-7-34

**Published:** 2008-07-02

**Authors:** Pascal Yaka, Benjamin Sultan, Hélène Broutin, Serge Janicot, Solenne Philippon, Nicole Fourquet

**Affiliations:** 1 ProdiG, UMR 8586, 2 rue Valette, 75005, Paris, France; 2 IRD – LOCEAN/IPSL (UR 182, UMR 7159 IRD-CNRS-UPMC) Université Pierre et Marie Curie, 4 place Jussieu, 75252, Paris, cedex 05, France; 3 G.E.M.I, UMR CNRS/IRD 2724, Equipe ≪ Evolution des Systèmes Symbiotiques ≫ Institut de Recherche pour le Développement (IRD), 911 Avenue Agropolis, BP 64501 34394, Montpellier ,Cedex 5, France; 4 Current address: Division of International Epidemiology and Population Studies, Fogarty International Center, National Institutes of Health, Bethesda, MD, USA

## Abstract

**Background:**

Every year, West Africa is afflicted with Meningococcal Meningitis (MCM) disease outbreaks. Although the seasonal and spatial patterns of disease cases have been shown to be linked to climate, the mechanisms responsible for these patterns are still not well identified.

**Results:**

A statistical analysis of annual incidence of MCM and climatic variables has been performed to highlight the relationships between climate and MCM for two highly afflicted countries: Niger and Burkina Faso. We found that disease resurgence in Niger and in Burkina Faso is likely to be partly controlled by the winter climate through enhanced Harmattan winds. Statistical models based only on climate indexes work well in Niger showing that 25% of the disease variance from year-to-year in this country can be explained by the winter climate but fail to represent accurately the disease dynamics in Burkina Faso.

**Conclusion:**

This study is an exploratory attempt to predict meningitis incidence by using only climate information. Although it points out significant statistical results it also stresses the difficulty of relating climate to interannual variability in meningitis outbreaks.

## Background

**Figure 1:**
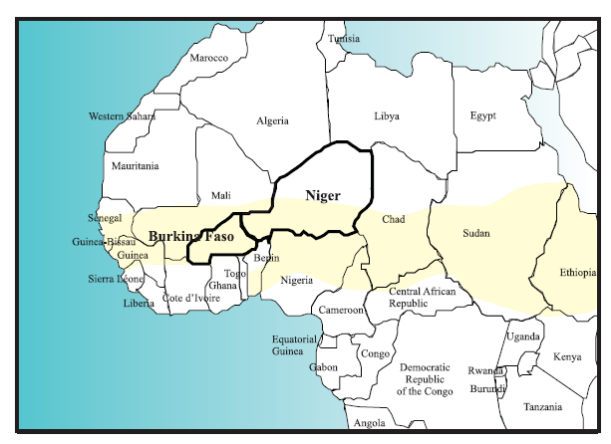
**Map of West Africa and the "Meningitis Belt"**. Modified from WHO (1998).

Meningococcal Meningitis (MCM) is a contagious infection disease due to the bacteria *Neisseria meningitis*. MCM epidemics occur worldwide but the highest incidence is observed in the "meningitis belt" (Fig. 1) of sub-Saharan Africa, stretching from Senegal to Ethiopia [1,2]. This region, first defined by Lapeyssonie in 1963 [2], is characterised by seasonal epidemics during the dry season which usually stop with the onset of rains, and also by large epidemics which occurred every 8–12 years, culminating in a massive epidemic in which nearly 200,000 cases were reported in 1996 [1,3]. Among the well-known different serotypes of *Neisseria meningitis *isolated in Africa such as serogroups A, C, Y and W135, group A remains the major serogroup responsible for African epidemics throughout the past 70 years [4] despite a recent emergence of serogroup W135 (see [5] for review).

The actual factors that initiate these epidemics are not yet clearly understood although we know that a complex interplay of social interactions [6], transmission of a new epidemic strain [4], susceptibility of populations [7], asymptomatic carriage [8] and environmental conditions [9] is involved. Among favourable conditions for the resurgence and dispersion of the disease, climatic conditions may be important inducing seasonal fluctuations in disease incidence and contributing to explain the spatial pattern of the disease roughly circumscribed to the ecological Sahelo-Sudanian band [2,10]. The role of climate on this meningitis seasonality and spatial distribution has been widely studied [11-13]. The Sahelo-Sudanian region is subjected to a sequence of dry winter, dominated by dry and dust-laden northern winds, called the Harmattan, and wet season starting at spring with the monsoon. A recent study has provided a clear, quantitative demonstration of the existing connections between meningococcal meningitis epidemics onset and the Harmattan winds [11]. The authors have shown a correlation between the week of the maximum speed of a Harmattan wind index and the week of the onset of the epidemics in Mali. Another recent study [14] found that anomalies in annual meningitis incidence at district level were related to monthly climate anomalies. Significant relationships were found for both estimates of dust and rainfall in the pre-, post- and epidemic season.

The objective of this study is twofold. First, we investigate the role of climate on the triggering of MCM epidemics by using a long-term dataset. Second, we explore the possibility to include the climate conditions as a predictor of meningitis epidemics. To do so, we start by defining an *a priori *hypothesis on the causal link between climate and disease. Based on a literature review, we assume that dry and windy weather conditions in early winter might cause damage to the mucous membranes of the respiratory system and/or inhibits mucosal immune facilitating the transfer of the bacterium to the meninges and thus create propitious conditions to the triggering of MCM epidemics [12,13,15]. According to this hypothesis, if the role of climate is strong enough, we should observe a positive correlation between the markers of these particular winter conditions (i.e. strong north-easterly wind, high pressure and dryness) and the MCM incidence. Since the triggering of epidemics is probably not only due to climate but results from numerous processes acting at different spatial hierarchical scales in various medical, demographical and socio-economical conditions, an absence of significant correlation between climate and disease would not necessary confound this hypothesis but could point out that climate is not a major driver. In order to verify our *a priori *hypothesis, we will examine the statistical links between winter climate variables and disease dynamics in two highly affected countries, Niger and Burkina Faso. Since most studies have focused on very small spatial scales, we will follow suggestions from Sultan et al. [11] and Broutin et al. [3] and use national-scale aggregated data. The aggregation of local data is a simple way to go beyond data heterogeneities and idiosyncratic details in order that only the important disease generalities, conditioned by the large-scale forcing, *e.g*. climate variability, remain.

## Materials and methods

### Epidemiological data

This work is based on the WHO disease surveillance over Africa. Details on the diagnosis used for meningitis can be found in WHO [16]. Meningitis reporting is incorporated into weekly reporting of notifiable diseases and aggregated at different spatial scales from the health unit to the country level. From this surveillance, WHO proposed a strategy aimed at early detection and control of meningitis epidemics at the level of health districts. This strategy is based on a strengthened epidemiological surveillance, mass immunization campaigns when incidence rate thresholds are exceeded and case management with appropriate antimicrobial therapy.

This database is available online on the WHO website and has already been used by Broutin et al. [3] to make a comparative study of meningitis dynamics across nine African countries. Although this dataset covers the period 1939–2005, we consider only the years after 1966 for two reasons. First, there is a large number of missing values before 1966. Second, Broutin et al. [3] have shown major changes after the 60's and the 70's in meningitis periodicities and synchronicities across African countries. These changes might be induced by the start of vaccinations at the end of 1970's in all of these countries. By taking into account only the post-vaccination period, we consider the most homogeneous time series to describe the meningitis dynamics but we have to keep in mind that a part of the variance of the incidence data might be explained by vaccination effect. However, as vaccination strategy has varied across country and within a same country (different proportions and with different types of vaccines, different policy from reactive to mass vaccination), the impact of vaccination is very difficult to point out in our time series.

In this study, in order to document the intensity of the disease from year to year and to work with a dataset as large as possible, we use the annual sum of MCM cases over eight African countries with one value per year and per country (Table 1; see Table 2 for the name of the eight countries). This annual sum of cases per country is not representative of the whole country but describes mainly the areas with the highest population. We perform two initial transformations of meningitis data before comparing them with climate data. First, since the MCM cases have a natural trend associated with population increase (which has more than trebled over the time period of investigation), we do not use directly the MCM cases but an incidence rate (latter IR) defined by the number of cases per 100.000 inhabitants (based on USGS total population estimates). The computation of this IR allows us to consider the thresholds defined by WHO for the epidemics surveillance. Secondly, since the meningitis data are highly skewed, as would be expected of an epidemic dataset, we apply a log-transform to IR (latter log-IR) in order to normalize the distribution.

**Table 1:** Summary of epidemiological and environmental datasets

	**Type of data**	**Available ** **data period**	**Used data** **period**	**Available time/space** **scale**	**Used time/space** **scale**
**1**	Meningitis cases	1939-today	1966–2005	Weekly/Health unit	Annual/Country
**2**	Environmental variables	1948-today	1968–2005	4 values per day/grid-spacing of 2.5° latitude by 2.5° longitude	Monthly/Country

The discrepancies between available and used data are justified in the Materials and Methods section.

**Table 2:** Classification of the MCM datasets for 8 West African countries

		**Very low** IR < 7.2	**Low** 7.2 < IR < 18.9	**High** 18.9 < IR < 41.3	**Very high** IR > 41.3
**1**	Benin	4	15	9	5
**2**	Burkina Faso	0	1	18	15
**3**	Chad	5	9	9	11
**4**	Mali	10	12	4	6
**5**	Niger	0	1	9	20
**6**	Nigeria	19	9	2	1
**7**	Sudan	18	2	6	4
**8**	Togo	8	14	7	2

Classification of the meningitis dataset (34 annual values from 1966 to 1999 for 8 West African countries) into four categories according to log-Incidence Rate (log-IR) thresholds. Since the log-IR are normally distributed, the classification has been done according to quartile groups which divide the sorted data set into four equal parts, so that each part represents 1/4 of the sampled population. The IR range of the four resulting categories is shown in the first row.

### Atmospheric data

The National Centers for Environmental Prediction (NCEP) and the National Center for Atmospheric Research (NCAR) have completed a reanalysis project with an up-to-date version of the Medium Range Forecast model [17]. This dataset consists in a reanalysis of the global observational network of meteorological variables and a forecast system to perform data assimilation throughout the period 1948 to present. However, prior to 1968 they are not fully reliable for the African continent, as demonstrated in Camberlin et al. [18].

We use these atmospheric variables over the period 1968–2005 in average with one value per month and per country (Table 1). Notice that for the case of Niger, the atmospheric variables are averaged not for the whole country as for Burkina Faso but only for the Southern half of Niger lying within the meningitis belt (Fig. 1). We select 7 variables (Table 3) that are likely to influence MCM disease outbreaks according to the literature:

**Table 3:** The environmental variables used in the study

	**Environmental variable**	**Code**
**1**	Zonal wind (m/s)	UWND
**2**	Meridional wind (m/s)	VWND
**3**	Wind speed (m/s)	MOD
**4**	Sea level pressure (Pa)	SLP
**5**	Surface temperature (°C)	AIR
**6**	Surface relative humidity (%)	RHUM
**7**	Surface specific humidity (kg/kg)	SHUM

- Wind velocity (zonal and meridional components, wind speed) and sea-level pressure that characterize the Harmattan circulation intensity. The influence of the latter circulation on meningitis has been shown recently [11].

- Surface temperature, specific and relative humidity near the surface that are markers of the dry conditions propitious to the MCM epidemics. The incidence of MCM has previously been correlated with dry and dusty conditions [1,9,13].

### Correspondence Analysis

**Figure 2:**
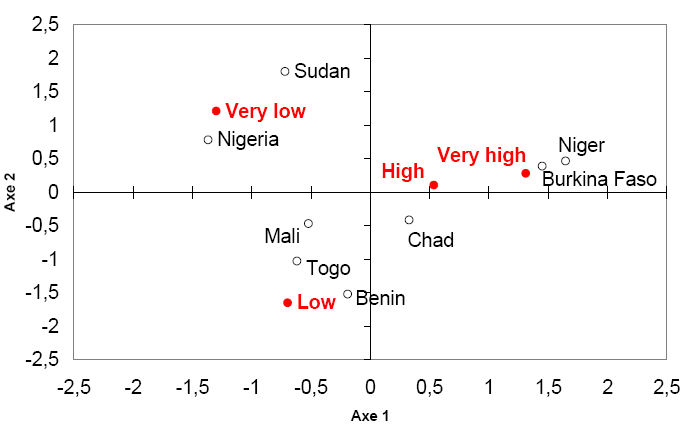
**The two main factors of the year-to-year disease variability in West Africa**. Graphical representation of the two main factors of the Correspondence Analysis where both rows (countries) and columns (four clusters characterizing the intensity of the epidemic) of the contingency table (Tab.2) are projected.

In order to summarize the MCM cases dataset composed of 34 annual values from 1966 to 1999 for each of the 8 West African countries (Table 1 and Table 2) under study and to compare the disease dynamics in these countries, we use the Correspondence Analysis (CA). This method is well-suited to look at the main structure of a normally distributed dataset without *a priori *hypothesis. Since we normalize the distribution of meningitis data by applying a log-transform to IR, this method can thus be applied to our data. For that:

- First, since the log-IR are normally distributed, it permits classification of the years according to quartile groups which divide the sorted data set into four equal parts so that each part represents 1/4 of the sampled population. For each country, we then classified the years of meningitis data into one of the four categories according to log-IR thresholds. The first 1/4 is defined as very low incidence group, the second is the "low incidence" group, the third is "high incidence" and the last one is the "very high" (Table 2). As a result, for Benin for instance, 4 years are considered with "very low" incidence, 15 years are "low", 9 are "high" and 5 are "very high".

- The IR ranges of the four resulting categories (Table 2) are pared to the thresholds defined by WHO for the epidemics surveillance. Two thresholds are used by WHO at the district level, the Alert Threshold (AT) which considers more than 5 cases per week and per 100.000 inhabitants and the Epidemic Threshold (ET) which is more than 10 cases per week and per 100.000 inhabitants. The lowest IR category regroups the years with less than 7.2 cases/100.000 inhabitants which is lower than the ET while the highest IR category regroups years with an IR four times greater than the ET. However, since data are used at a more aggregated space and time scale (country instead of district and year instead of week), there is no direct correspondence between these thresholds and the quartiles.

- We then construct a two-way contingency table from the original dataset which is a tabular cross-classification of data such that one subcategory (countries) is indicated in rows and another subcategory (the four IR categories) is indicated in columns (Table 2).

- Finally, we apply a CA which is able to give the best simultaneous representation of the rows and the columns of such two-way contingency table. CA is based on the extraction of the principal canonical correlations and corresponding row and column-scores from a correspondence analysis of a two-way contingency table [19]. From the row and column-scores on the two main factors, it is possible to make a graphical representation of both rows (countries) and columns (the four IR categories) of the contingency table (see Fig. 2). Such graphical representation gives a good summary of the structure of the original dataset highlighting proximities between one country and another and between countries and IR categories. Notice that the distance between two countries is not driven by the average IR over the period 1966–1999 but by the number of years in each cluster. We thus are able to compare the distribution of MCM annual IR for each country.

### Composite analysis

In order to detect relationships between climate and MCM incidence, we perform a composite analysis. For each country, we first classify the years into two sub-groups: the years with the highest IR (the years with a log-IR greater than the third quartile) and the years with the lowest IR (the years with a log-IR lower than the first quartile). We then average separately the atmospheric data for the high (HIGH) and low (LOW) MCM incidence years. Finally we compute the HIGH minus LOW difference in order to point out the atmospheric situations characterizing a typical high incidence year. The significance of this difference is attested using a Student test.

### Correlations between climate and MCM

In order to detect relationships between climate and MCM incidence, we compute the correlations between the monthly atmospheric variables and the annual log-IR. For each country, we compute the correlation coefficients between each of the 7 atmospheric values for one month and the annual log-IR of the country. These correlations are given for the 4 fall-winter months: October to January. They are computed over 1968–2005. Because of the number of variables used for the correlations computation (7 variables over 4 months), there is always the possibility of a chance association emerging. Classical significance tests do not consider this risk since there are applied independently for each time series and do not take into account the repetition of the correlation computation which increases the possibility of a chance association. We thus set-up a test reproducing our experimental conditions: we generate a set of 7 × 4 random Gaussian time series with the same properties (mean and variance) than the atmospheric variables and compute the correlation between each random time series and the log-IR of the considered country. We then use to assess the significance of the correlations at the 1% level of confidence the threshold given by the positive (negative) correlation values of the 99% (1%) quantile of the 7 × 4 random correlation values. This experiment is reproduced 10, 000 times and we average the positive and negative thresholds over the 10, 000 repetitions.

### Multivariate linear regression models

The analysis of the links between climate and MCM are then used to select a set of atmospheric predictors that likely influence the MCM incidence. These predictors can be used to build up a stepwise multivariate linear regression model in order to predict the annual MCM incidence rate. The equation of the multivariate model is:

*Y *= α + β_1_*X*_1 _+ ⋯ + β_*p*_*X*_*p*_

*Y *represents the predicted log-IR value, α is a constant, and each β terms denotes a regression coefficient for the corresponding predictor *X*.

The robustness and the forecast skill of the regression models are assessed using two standard methods: the cross-validated correlation and the Relative Operating Characteristics (ROC) score:

- A simple leave-one-out cross-validation is used to document the stability of the regression models: we compute the model parameters from a portion of the data (called training period) composed by all years minus one and we look at the prediction of the remaining data. The cross-validated correlation is a much more realistic representation of the skill of the model applied to "unseen" years.

- ROC is a means of testing the skill of categorical forecasts [20,21]. It is based on contingency tables giving the Hit Rate (HR) and False Alarm Rate (FAR). We first transform our data and forecasts into binary time series where only two outcomes are possible, an occurrence of high incidence year or a non-occurrence, according if the log-IR of the year is greater or above the median which divides the dataset in half. We then compute a contingency table based on these two categories and calculate the HR and FAR which are simply percentages that tell us how well the forecast did when a high incidence year was observed, and likewise, how well the forecast did when a high incidence year was not observed. An example of this contingency table is given in Table 4. The "hits" ("zeros") category represents the number of high (non-high) incidence rate that have been forecasted as so. The "false alarms" ("misses") category represents the number of non-high (high) incidence years that have been forecasted as high (non-high) incidence years. The HR is defined as:

**Table 4:** Hits and False alarms in forecast models

		**Predicted**	
		
		**Non-High ** **incidence year**	**High ** **incidence year**
**Observed**	**Non-High ** **incidence year**	zeros	false alarms
	**High ** **incidence year**	Misses	hits

Illustration of the contingency table used to evaluate the forecast models. We first transform our data and forecasts into binary time series where only two outcomes are possible, an occurrence of high incidence year or a non-occurrence, according if the log-IR of the year is greater or above the median which divides the dataset in half. We then compute a contingency table based on these two categories. The "hits" ("zeros") category represents the number of high (non-high) incidence rate that have been forecasted as so. The "false alarms" ("misses") category represents the number of non-high (high) incidence years that have been forecasted as high (non-high) incidence years.

$$HR=\frac{hits}{hits+misses}$$

It is comprised between 0 and 1, 1 meaning that all occurrences of high incidence year were correctly forecast as so. The FAR is defined as:

$$FAR=\frac{false\;alarms}{hits+false\;alarms}$$

It is comprised between 0 and 1, 0 meaning that all forecasted high incidence year were observed as so. The ROC score is a measure of the hit rate to false-alarm rate. It is recognised that the ROC score as applied to deterministic forecasts is equivalent to the scaled Hanssen and Kuipers score (HKS; 21). HKS is defined as:

$$HKS=\frac{HR-FAR+1}{2}$$

The range of possible values goes from 0 to 1 where a perfect forecast system has a value of one and a forecast system with no information has an value of 0.5 (HR being equal to FAR).

In our study, these standard forecast skill methods are also applied to a reference forecast method based on persistence (the incidence rate of one year is the same than the incidence rate of the previous year). Since the persistence is the simplest way to produce a forecast, we consider the skill of our regression models as useful if it is greater than the persistence skill.

### Susceptible-Infected Model

In order to illustrate hypotheses on the disease transmission, we use a Susceptible-Infected (SI) model. Such models [22] are widely used for direct infectious disease in order to examine transmission processes [23]. It consists of two compartments: Susceptible (S) and Infected (I). Individuals in the S compartment are susceptible to be infected and move to the I compartment with a speed controlled by a transmission rate. The initial sizes of the two compartments and the transmission rate are the parameters needed to fit the model. We choose these parameters to reproduce roughly the same temporal characteristics of an outbreak in Burkina Faso. To do so, we use a discrete model and simulate an outbreak over one virtual year of 52 time steps (a weekly time step) with a peak occurring during the first half of the year. Notice that the SI model is mainly used here for an illustrative purpose.

## Results

The year-to-year variability of meningitis cases and incidence rates is described for 8 West African countries over the 34-year period between 1966 and 1999. The CA is used to compare the disease dynamics in these countries (see Materials and Methods). Figure 2 represents graphically the 8 countries in the two main factors of the CA applied to the MCM incidence rates. The proximity between countries is driven by the number of years in the 4 incidence categories (Table 2). The main factor (the horizontal axis) discriminates (i) the countries with a high number of low MCM incidence years (i.e. Sudan, Nigeria) on the left side of the axis and (ii) the highly affected countries with the greatest number of high MCM incidence years (Burkina Faso and Niger) on the right side of the axis. The second factor (the vertical axis) isolates the countries with a large number of years whose annual MCM incidence rates are close to the 1966–1999 average (high and low, i.e. Mali, Togo, Chad and Benin). The analysis thus points out three different disease dynamics in these countries: the "high-risk" countries characterized by several years with very severe epidemics, the "medium-risk" countries mainly with MCM cases each year but without severe epidemics, the "low-risk" countries with very low MCM cases each year. We now focus our study on the two "high-risk" countries, i.e. Niger and Burkina Faso to analyze the link between MCM incidence and climate.

**Figure 3:**
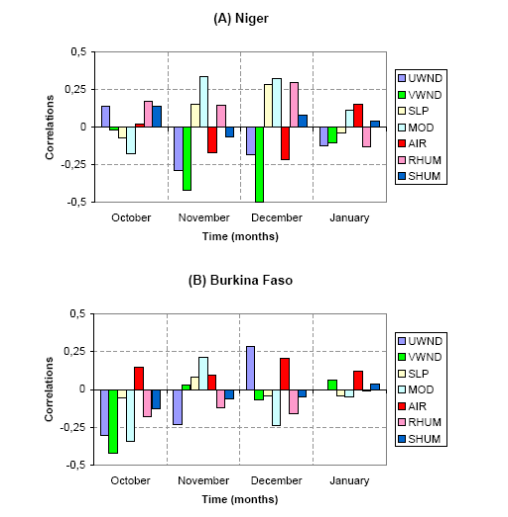
**Correlations between climate and meningitis in Niger and Burkina Faso**. (A) Correlation coefficients between the MCM annual log-incidence rates in Niger and 7 monthly averaged atmospheric variables over Niger. The correlations are computed for each month from October to January over the 1968–2005 period. The monthly atmospheric variables from October to December of the year *y *are correlated with the MCM log-incidence rate of the following year *y+1*, while the monthly atmospheric variables in January of the year *y *are correlated with the incidence MCM rate of the same year *y*. Significant values at the 99% confidence interval are outside of the shaded box (see Materials and Methods for details on the significance test). The atmospheric variables (Tab.3) are: UWND the zonal wind, VWND the meridional wind, SLP the seal-level pressure, MOD the wind speed, AIR the surface temperature, RHUM the relative humidity and SHUM the specific humidity. (B) Same as (A) but for atmospheric variables and MCM incidence in Burkina Faso.

**Figure 4:**
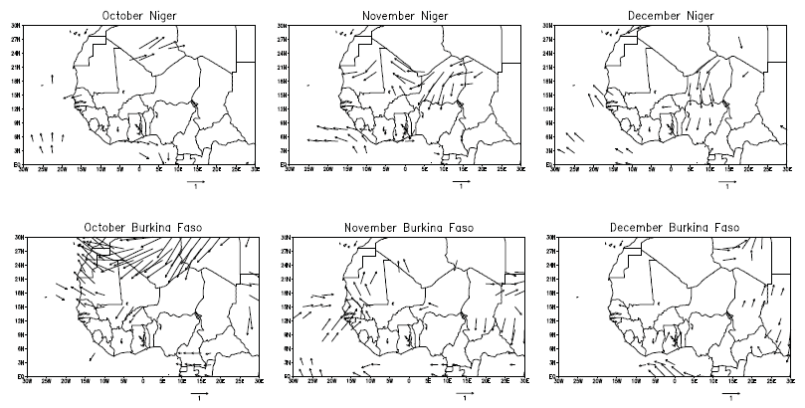
**The atmospheric situations characterizing a typical high incidence year**. A composite analysis is performed by averaging separately the surface wind data for the years recording the highest (HIGH) and the lowest (LOW) incidence rates in Niger (top) and in Burkina Faso. The maps show the HIGH minus LOW difference in the October, November and December wind data (m/s) in order to point out the atmospheric situations characterizing a typical high incidence year. Only significant values at the 10% confidence interval using a Student test are reported.

In order to point out the impact of climate on the triggering of MCM epidemics in Niger and Burkina Faso, we calculate the correlations between winter climate variables and the MCM annual log-IR over the period 1968–2005 to verify the *a priori *hypothesis defined in the background section. Correlations are computed for 4 months from October to January in order to focus our study on the atmospheric dynamics before the onset and the seasonal maximum of MCM cases [11]. Figure 3 represents graphically these correlations for both Niger (top) and Burkina Faso (bottom). Even if the correlations values are low, persistent and significant (at the 99% confidence interval; see Materials and Methods for the detail of the significance test) correlations between climate and MCM are found in Niger. We observe in November and in December a negative correlation between MCM incidence and the meridional wind component. As a negative value of the meridional wind component depicts a northerly wind, this correlation pattern is coherent with our hypothesis associating an enhancement of the Harmattan flow with an increase of MCM incidence. However the climate signal is less clear in Burkina Faso where the only significant values (at the 99% confidence interval) are found in October. As for Niger, we observe a negative correlation between the MCM incidence and the meridional wind component implying an enhancement of the northerly Harmattan flow. In order to attest the robustness of these correlations and to document their spatial extension, we perform a composite analysis by averaging separately the wind data for the years recording the highest (HIGH) and the lowest (LOW) incidence rates. Figure 4 shows the HIGH minus LOW difference in the October, November and December wind data in order to point out the atmospheric situations characterizing a typical high incidence year. Only significant values at the 10% confidence interval are reported in Fig. 4. The composite analysis in Niger (top of the Fig. 4) shows a very clear enhancement of the Harmattan winds in November and December consistent with the correlations results. This enhancement of the north-easterly flow is mainly limited to Niger attesting the robustness of the results. However this enhancement does not persist in January and later in the year (not shown). The HIGH minus LOW difference is less clear in Burkina Faso. Only the atmospheric situation in October shows a difference pattern coherent with our *a priori *hypothesis, pointing out a local increase of the north-easterly flow in Burkina Faso and an enhancement of a large anticyclonic anomaly in northern Africa favourable to the strengthening of the Harmattan wind.

**Figure 5:**
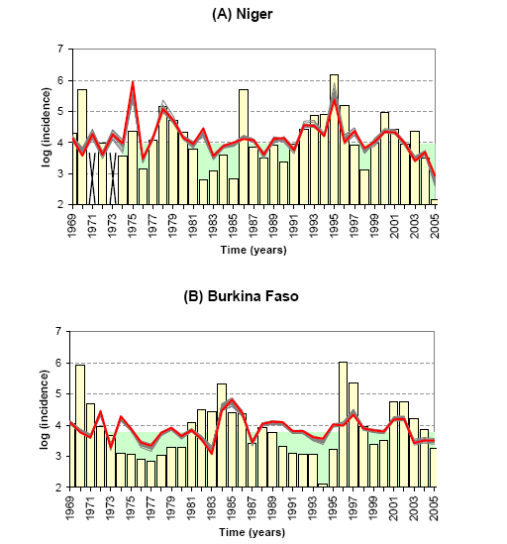
**Predictions of meningitis outbreaks in Niger and Burkina Faso**. Observed (yellow) MCM annual log-incidence time series and cross-validated forecast (red) for Niger (A) and Burkina Faso (B) from 1968 to 2005. The grey lines represent each individual forecast produced during the cross-validation process. The values in the green area correspond to the values lower than the median and have been considered as low incidence years in the ROC process. Alternatively the values greater than the median have been considered as high incidence years. The crosses depict missing values.

Based on the significant correlations we previously obtained between climate and disease, we explore the predictability of the disease in Niger and in Burkina Faso by using winter climate indexes. For each country, we select a set of atmospheric predictors among the atmospheric variables showing the clearest interpretative link with the annual incidence of MCM. These predictors are used to build a step-wise multivariate regression in order to predict the annual MCM log-IR separately in Niger and in Burkina Faso (see Material and Methods). Among the atmospheric variables averaged over Niger, we retain two predictors for the Niger model, the meridional wind components both in November and December. Among the atmospheric variables averaged over Burkina Faso, we retain one predictor in Burkina Faso, the meridional wind component in October. The results of the two models are shown in Figure 5 and Table 5. The correlation between the observed and predicted MCM time series is encouraging in Niger up to 0.62, but lower in Burkina Faso (R = 0.42). The decadal variability of the disease is well predicted by the model as well as several high values in Niger such as 1978 and 1995. However the Niger model misses several strong epidemic years (see 1970 and 1986). The robustness and the skill of the two models can be attested by the calculation of the Cross-Validated Correlation, the False Alarm Rate (FAR), the Hit Rate and the Hanssen and Kuipers score (respectively CVC, FAR, HR and HKS; see Materials and Methods). Table 5 gives the CVC, FAR, HR and HKS values for the Niger and Burkina Faso models and compares these values to the skill scores obtained by using persistence to forecast the incidence rate (the incidence rate of one year is the same than the incidence rate of the previous year). The CVC is 0.50 for the Niger model which is less than the correlation given above but still significant and greater than the persistence forecast skill. The squared CVC is around 0.25 which means that 25% of the disease variance from year-to-year is explained by the climate variability in winter. The cross-validated prediction (the red line in Fig. 5) tends to under-estimate the extreme values of the MCM incidence rates (see 1995 for the Niger model). The HR of the Niger model is high (HR = 0.83) and better than the persistence HR (HR = 0.67) meaning that 83% of the high incidence years have been forecasted as so. However the Niger model tends to overestimate the number of high incidence years with a FAR greater than the persistence FAR (0 is being desirable). By considering both good and bad forecasts using HKS, it appears than the Niger model has a predictive value since the HKS is greater than 0.5 (HKS = 0.68). The skill score is greater for the Niger model than for a persistence-based prediction but the difference is not very large (HKS = 0.63 for the persistence model).

**Table 5:** Skill scores of the meningitis forecast models

	**Niger**		**Burkina Faso**	
		
	Climate-based model	Persistence model	Climate-based model	Persistence model
CVC	0.50	0.30	0.33	0.55
HR	0.83	0.67	0.74	0.79
FAR	0.47	0.41	0.50	0.28
HKS	0.68	0.63	0.62	0.76

The skill is given by the Cross-Validated Correlation (CVC; 1 being desirable), the Hit Rate (HR; 1 being desirable), the False Alarm Rate (FAR; 0 being desirable) and the scaled Hanssen and Kuipers score (HKS; 1 being desirable). The skill of the climate-based model developed in this study is compared to the reference skill of a persistence-based model (the incidence rate of one year is the same than the incidence rate of the previous year). Since the persistence is the simplest way to produce a forecast, we consider the skill of our climate-based models as useful if it is greater than the persistence skill.

The Burkina Faso model gives lower scores with a correlation between the observed and predicted MCM time series around 0.42 and falling to 0.33 for the CVC. Forecasts based on persistence do a better job for the four skill scores CVC, HR, FAR and HKS (see Table 5). These low skill scores can be partly explained by the fact that only one predictor in the atmospheric circulation could be found to build the regression model while two predictors were used in Niger.

## Discussion

In this study, the relationships between climate and MCM disease at interannual and country scales have been statistically investigated in two highly affected countries: Niger and Burkina Faso. We pointed that these links are particularly clear in Niger and weak but significant in Burkina Faso. The disease resurgences in Niger and in Burkina Faso are linked with an enhancement of the winter conditions, e.g. enhanced Harmattan winds over Niger in November/December and over Burkina Faso in October. These findings are coherent with a previous study which showed a positive correlation between the October dust and meningitis incidence in Burkina Faso, Mali and Niger [14]. Here, we also defined relevant climatic variables for the construction of linear models to forecast MCM epidemics intensity from year to year. These statistical models work well for Niger showing that 25% of the disease variance from year-to-year in this country can be explained by the winter climate but fail to represent accurately the disease dynamics in Burkina Faso. Although this study points out significant statistical results, it also stresses the difficulty of relating climate to interannual variability in meningitis outbreaks. Numerous reasons can be pointed out to explain this limitation but two are of most importance:

First, the final size of the outbreak clearly does not depend only on climate but implies many other factors. The size of the epidemics will be also (and perhaps mainly) driven by the immunity of the affected population against the serotype involved in the outbreak and socio-economic factors (pilgrimages, migrations) [6,7]. The proportion of carriers might also play an important role in the disease dynamics (see [8] for review). Vaccination, even if there is a debate on the efficiency on the meningitis control activities [24-26], has certainly an impact on the final size of the outbreak as it is suggested by Broutin et al. [3] in their comparative studies of meningitis dynamics across several African countries. The second important limitation to our study corresponds to the meningitis data themselves. Missing values as well as suspected underreporting may introduce some biases in the incidence time series. As a consequence of the above factors, influencing the final size of the outbreak, it is thus very likely that the meningitis incidence data contain trends, strong or low incidence events or periods that can not be related to any climate effect. It is probably what starts to account for the differences in results between countries. The weak correlation between climate and disease in Burkina Faso does not necessary confound our hypothesis of the dry northerly winds being implicated in the outbreaks of meningitis but could point out that climate is not a major driver of the disease dynamics. Alternatively, since the variability of the meningitis incidence from one year to another results from numerous processes acting at different spatial hierarchical scales in various medical, demographical and socio-economical conditions, the results obtained with the Niger model, even if the correlation values and the explained variance are weak, suggest that climate is an important driver for the triggering of epidemics in Niger and validate the large-scale approach that allows to smooth local data heterogeneities.

**Figure 6:**
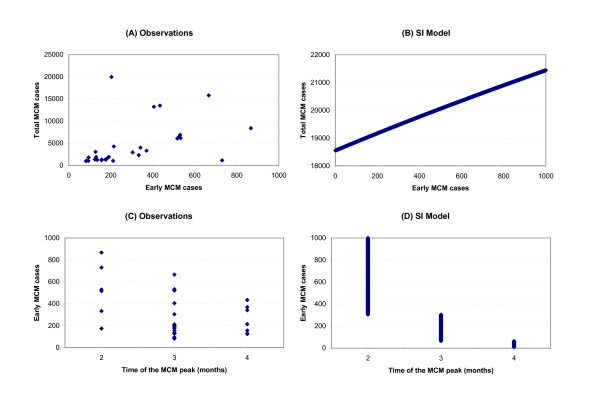
**The importance of early cases in Burkina Faso and in a basic Susceptible-Infected model**. (Left) A – Relationship between the number of cases during the peak months (from January to April) and the number of early cases (from October and December). C – Relationship between the number of early cases (from October and December) and the timing of the peak month (the month with the highest number of cases). To produce the panels A and C, we use monthly cases in Burkina Faso from 1961 to 2005 (the longest complete monthly time series). (Right) B – Sensitivity of the final number of infected individuals to the increase of the initial number of infected individuals in a basic Susceptible-Infected (SI) model. D – Relationship between the initial number of infected individuals and the timing of the peak month (the month with the highest number of cases) in the SI model. The SI model is mainly used here for an illustrative purpose.

Moreover, such statistical study can only demonstrate a statistically significant association and not causation. It is always possible that changes in climate are linked to other factors, such as a change in social behaviour that is the key determinant of the effect. This point is of importance as there is no robust physiological mechanism for the role of climate in disease occurrence. In our study and in that of Thomson et al. [14], the correlations between climate and disease are depicted very early in the meningitis epidemic season and do not persist during the epidemic season. It is not clear how atmospheric conditions in October-November-December (OND) affect an outbreak occurring 3–4 months later since the incubation period of meningococcal meningitis is a few day to weeks. Different climate influences with longer and shorter relationships to disease incidence may be at work to explain these correlations. On one hand, climate could have a cumulative effect on the vulnerability of the population to the infection. Long-term exposure to air dryness and strong dust winds might weaken resistance of human oro-pharyngeal membranes through successive respiratory infections making propitious conditions to the passage and inner release of the bacteria responsible for the disease when the organism is carried [15]. On the other hand OND winter conditions could act with a shorter time-lag by enhancing meningococcal invasiveness during the pre-epidemic season through the same mechanism explained above, i.e. direct damage of the mucosal barrier and/or an inhibition of the mucosal immune defences [15]. The early cases induced by climate effect could then have an influence on the final size of the outbreak through contacts within and among households and communities since these contacts increase the risk of acquiring infection [27] and increase the carrier rate of the pathogenic serogroup (see [8] for review). The importance of early cases in the final size of the outbreak has already been stressed by WHO [28] that consider early cases in the season as a warning sign of large epidemic. This importance is illustrated by Figure 6 using monthly cases in Burkina Faso from 1961 to 2005 (the longest complete monthly time series). Fig. 6a shows that the number of cases during the peak months (January to April) increases with the number of early cases occurring between October and December. Another interesting point is that early cases seem to impact the seasonal cycle of meningitis with the peak month of the epidemic being advanced (delayed) with a high (low) number of early cases (Fig. 6c). This increase of the epidemic size combined with the advance of the epidemic peak is a well known response of basic SI epidemiological models to the increase of the initial number of infected individuals. Fig. 6 shows the response to an increase of initial infected individuals in a SI model fitted to reproduce roughly the same temporal characteristics of an outbreak in Burkina Faso. Notice that the response of the SI model to the initial infected cases does not depend on the value of its parameters (i.e., the initial size of the susceptible population and the transmission rate). The similarity of Fig. 6a,c and Fig. 6b,d could suggest an importance of transmission in the final size of the outbreak. In the latter hypothesis, climate would act indirectly in transmission by increasing the number of infected hosts at the beginning of the epidemic season when the reproductive ratio is very low; this increase being responsible, trough transmission, in the increase of the final size of epidemics. However even if the disease might play a role in transmission [27], meningococcal transmission often occurs in the absence of a corresponding increase of the rate of meningococcal disease, through asymptomatic carriers, indicating that transmission alone is not sufficient to trigger an epidemic [13,15].

Given these difficulties, much more work need to be done to use such climate indexes in the context of a survey and an early warning system (EWS) to influence public health policy, for example setting in place an epidemic preparedness programme, ordering vaccine, etc. After this work, it is clear that the opportunity of the use of environmental variables in such EWS should now be further investigated by including other factors which also are likely important drivers in MCM dynamics. In particular, epidemiological and population parameters (immunity, carriers, population size, ...) should clearly be including in any forecasting tool as well as behaviour factors like migrations. This approach will remain explorative since the main risk and control factors for the disease and how they interplay each other are not better understood. Further combined epidemiological and climate studies are recommended to help in a better understanding of MCM dynamics and evolution at different spatial-scales. A key issue is the extent and the improvement of epidemiological and environmental datasets through long-term longitudinal studies (see for instance [7] for the immunological factors) and collection of both environmental and epidemiological data over the same site [29]. These improvements are necessary but might not be sufficient to provide highly precise forecasts since stochastic processes in the transmission could limit the predictability of the final size of the outbreak. The limits to the precision of EWS for epidemics of infectious disease have been discussed recently by Drake [23]. According to the author, the characteristics of emerging diseases to which human populations are highly susceptible prevent precise forecasts because of the micro-scale component (contacts within and among households and communities) whose small variations could induce large variations in the final size of the outbreak. While the forecast of the EWS based on climate and other environmental characteristics contain only the macro-scale source of variation and not the micro-scale causes, they can still be used effectively to define risk indicators rather than precise forecasts that could be used to better control MCM disease.

## Authors' contributions

PY and BS conceived the study, participated in its design, carried out the acquisition, analysis, and interpretation of data, and drafted the first version of the manuscript. SP contributed to process epidemiological data and to interpret the results. HB participated in interpretation of the results and the writing of the paper. SJ and NF supervised and participated in all phases of the study. All authors read and approved the final manuscript.
